# Comprehensive analysis of adverse events associated with onasemnogene abeparvovec (Zolgensma) in spinal muscular atrophy patients: insights from FAERS database

**DOI:** 10.3389/fphar.2024.1475884

**Published:** 2025-01-07

**Authors:** Wenwen Zhang, Yizhen Yin, Dan Yang, Mengyuan Liu, Caixia Ye, Ruiling Yan, Ruiman Li

**Affiliations:** Department of Fetal Medicine, The First Affiliated Hospital of Jinan University, Guangzhou, China

**Keywords:** Zolgensma, gene therapy, FAERS, spinal muscular atrophy, adverse drug event

## Abstract

Onasemnogene Abeparvovec (Zolgensma) is a gene therapy for the treatment of Spinal Muscular Atrophy (SMA) with improved motor neuron function and the potential for a singular treatment. Information on its adverse drug reactions is mainly from clinical trials and real-world studies with extensive sample sizes are lacking. In this study, we analyzed the U.S. Food and Drug Administration’s Adverse Event Reporting System (FAERS) database to assess the drug safety profile of Zolgensma. A total of 1951 adverse event reports associated with onasemnogene abeparvovec (Zolgensma), containing 778 import important medical event (IME) signals, were identified from the FAERS database, and multiple disproportionate analysis algorithms were used to determine the significance of these adverse events. This study identified 281 onasemnogene abeparvovec-related adverse events (AEs), including some significant adverse events not mentioned in the product labelling. Elevated liver enzymes, fever, vomiting, and thrombocytopenia were the most common adverse reactions. Most adverse events manifested within the initial month of onasemnogene abeparvovec use, especially the first 8 days, but some may still occur after 1 year of treatment. Sex-specific scrutiny revealed differing risk levels for adverse events among women and men. Thrombocytopenia and thrombotic microangiopathy are more common in patients weighing ≥8.5 kg, and changes in renal function need to be closely monitored if thrombotic microangiopathy occurs. The above findings provide valuable insights into optimizing the utilization of onasemnogene abeparvovec, improving its effectiveness, and minimizing potential side effects, thereby greatly facilitating its practical application in clinical settings.

## 1 Introduction

Spinal muscular atrophy (SMA) is a rare genetic neuromuscular disorder characterized by progressive muscle wasting (atrophy) and weakness that results in loss of movement. Untreated SMA is often cited as the primary genetic cause of death in young children (Chaytow et al., 2018; Schorling et al., 2020). The exact global prevalence of SMA is uncertain and various depending on the types of the condition (Prior et al., 2020; Torricelli, 2022). In the United States and the European Union, it is estimated that there are 30,000–35,000 cases of SMA (Yeo et al., 2024), with an overall incidence of approximately 1/6,000 to 1/10,000 births (Arnold et al., 2015; Kichula et al., 2021; Day et al., 2022; Nishio et al., 2023). SMA is mostly caused by homozygous loss-of-function (LOF) mutations in the survival motor neuron 1 (SMN1) gene located on the long arm of human chromosome 5 (5q13.2). The SMN protein is a widely expressed RNA-binding protein that plays a critical role in various cellular processes, including the assembly of snRNP complexes in the cytoplasm, RNA metabolism, DNA repair, and the maintenance of cellular homeostasis. Currently, therapeutic strategies for SMA mainly focus on increasing the expression levels of the SMN protein. It is noteworthy that there is a highly homologous copy of the SMN1 gene in humans, known as the SMN2 gene. The SMN2 gene produces extremely low levels of SMN protein from its transcripts due to the presence of a C to T point mutation in exon 7 (specifically C.840C>T). This mutation renders the mRNA produced by the SMN2 gene unstable and prone to degradation. Therefore, the severity of SMA is negatively correlated with the expression levels of the SMN protein and the copy number of the SMN2 gene (Feldkötter et al., 2002; Chen et al., 2020).

Onasemnogene abeparvovec (Zolgensma) is an adeno-associated virus (AAV) 9-based gene delivery system using a cDNA version of the SMN1 gene. This system has been developed to address the challenges posed by biallelic deletions or pathogenic variants of the SMN1 gene, which can lead to spinal muscular atrophy (SMA) (Xie et al., 2024; Rhee et al., 2024). This drug can penetrate the blood-brain barrier, and the AAV vector translocates into the nucleus, where the transgene acts as an episome, a small stale chromosome separates from the native chromosome, but does not integrate into the host DNA after entering the host cell. A single dose of 1.1 × 10^1^⁴ vector genomes per kg of intravenous Zolgensma was approved by the Food and Drug Administration (FDA) in May, 2019, as a one-time treatment for patients under 2 years of age with spinal muscular atrophy (Novartis Gene Therapies, 2024). This drug is a one-time treatment given through an intravenous infusion that takes 1 h. Clinical trials and real-world evidence indicates that treatment with intravenous onasemnogene abeparvovec improves motor function in children with spinal muscular atrophy aged 24 months or younger, but acute liver dysfunction can sometimes be severe and require extended prednisolone treatment. Post-treatment blood work monitoring shows hematologic abnormalities (decreased platelet counts and thrombocytopenia) and signs of hepatotoxicity (increased levels of transaminases) following Zolgensma administration. Moreover, patients with spinal muscular atrophy type 1 might still need respiratory and nutritional support after receiving Zolgensma (Day et al., 2021b; Stevens et al., 2020; Kotulska et al., 2021; Favia et al., 2024; Blair, 2022; Ogbonmide et al., 2023).

Considering the widespread clinical use of onasemnogene abeparvovec (Zolgensma), it is unclear whether other serious AEs may exist and what long-term complications may arise in individuals receiving early and/or presymptomatic targeted therapy. Hence, it is necessary to perform a comprehensive analysis of AEs associated with onasemnogene abeparvovec (Zolgensma) to detect the potential signals. As a spontaneous reporting system, the US FDA Adverse Event Reporting System (FAERS) database is widely used in pharmacovigilance analysis. Data mining of case reports from the FAERS database can provide us with valuable information about the safety of specific drugs in the real world (Liao et al., 2021; Yang et al., 2021; Katsuhara and Ikeda, 2021). In this study, we conducted a comprehensive disproportionality analysis of AEs and important medical events (IMEs) related to Zolgensma using the FAERS database. IMEs are a list of terms developed by the European Medicines Agency (EMA) designed to help prioritize the review of serious adverse drug reactions (ADRs) reported in suspected drug ADR reports, including those that result in death, are life-threatening, require hospitalization or prolong hospitalization, lead to persistent or significant disability/functional impairment, and involve congenital anomalies/birth defects (EudraVigilance system overview (EMA, 2016). The medical significance of these events lies in the fact that, although they may not immediately threaten life, cause death, or require hospitalization, they can have serious consequences if preventive medical measures are not taken (EudraVigilance system overview (EMA, 2016). Mining the FAERS database provides us with valuable information about the safety of onasemnogene abeparvovec in the real world, which is crucial for detecting potential signals, determining the statistical association between drug exposure and specific adverse events, and raising further attention and research on this issue.

## 2 Materials and methods

### 2.1 Data source and collection

We launched a pharmacovigilance study of onasemnogene abeparvovec in the post-marketing setting utilising data spanning the period from the second quarter of 2019 to the first quarter of 2024, sourced from the FDA Adverse Event Reporting System (FAERS) database. The FAERS database is based on the International Safety Reporting Guidelines (ICH E2B) established by International Conference on Harmonization (ICH), with adverse events categorized according to the Medical Dictionary for Regulatory Activities (MedDRA, version 26.1). The FAERS data files are comprised of seven distinct databases, including demographic and administrative information (DEMO), adverse drug reaction information (REAC), patient outcome information (OUTC), drug information (DRUG), drug therapy starts dates and end dates (THER), information on report sources (RPSR), and indications for use/diagnosis (INDI). The drug codes reported in the drug information include primary suspect (PS), secondary suspect drug (SS), concomitant (C), and interacting (I). Duplicate reports were identified and removed by selecting the latest FDA_DT (date the FDA picked up the case) with the same CASEID (FAERS case identification number) or, when the CASEID and FDA_DT were identical, the higher PRIMARYID (a unique identifier for FAERS reports). Throughout the study period, totally 8,838,535 reports related to onasemnogene abeparvovec were extracted from the FAERS database. 7,709,978 case reports of onasemnogene abeparvovec as the PS drug after the exclusion of duplicates, and 1,951 AEs were associated with onasemnogene abeparvovec (Supplementary Figure S1). All AEs reports of onasemnogene abeparvovec were identified in system organ class (SOC) and preferred term (PT) levels. Moreover, the generic name (onasemnogene abeparvovec-xioi), trade name (ZOLGENSMA), and Research and Development Code (AVXS-101, OAV-101) were defined as target drugs in the DRUG file.

### 2.2 Statistical analysis

The association between onasemnogene abeparvovec (Zolgensma) and AEs was determined by four algorithms: the reporting odds ratio (ROR), the proportional reporting ratio (PRR), the Bayesian confidence propagation neural network (BCPNN) and the multi-item gamma Poisson shrinker (MGPS) algorithm, which relied on disproportionality analysis (Shu et al., 2022a; Yin et al., 2022). The equations and criteria for the four algorithms are outlined in Supplementary Table S1. The data selected for analysis in this study were adverse event (AE) signals that met the four algorithm standards. Novelty signals were identified as any significant AE not listed in package inserts (Shu et al., 2022b).

The time to onset of onasemnogene abeparvovec AEs was defined as the interval between EVENT_DT (date of onset of AEs, in the DEMO file) and START_DT (date of initiation of onasemnogene abeparvovec, in the THER file). Deleted data include inaccurate or missing dates and EVENT_DT being earlier than START_DT. Microsoft EXCEL 2019, R software (version 4.3.3) was mainly used for data processing and analysis. The “ggplot2” package in the R software was used for data visualisation.

## 3 Results

### 3.1 General characteristic

From the second quarter of 2019 to the first quarter of 2024, a total of 1951 AEs were documented in the FAERS database, with 1951 records involving onasemnogene abeparvovec monotherapy, of which 778 cases were IMEs. The clinical characteristics of onasemnogene abeparvovec-associated AEs were described in Table 1. For sex, the incidence of AEs in females (45.70% for AEs, 48.46% for IMEs) accounted for a larger proportion than males (40.00% for AEs, 38.43% for IMEs). In terms of age composition, patients whose age were between 6 and 18 months accounted for a higher proportion (24.91%) than patients under 6 months and patients whose age over 18 months old. US (55.90% for AEs, 35.86% for IMEs) reported the largest number of AEs, followed by Russian (4.30% for AEs, 5.66% for IMEs), United Kingdom (3.20% for AEs, 5.53% for IMEs), Japan (3.10% for AEs, 4.5% for IMEs), and Germany (2.60% for AEs, 4.37% for IMEs). Serious outcomes include death, life-threatening, hospitalization, disability, and other serious outcomes. We divide one of them by all serious outcomes’ reports, to get the proportion. Hospitalization (22.96%) was the most frequently reported serious outcome which might be related to disease progression. Other serious outcomes and death were reported in 441 (22.60%) and 110 (5.64%) cases, respectively. Excluding the unknown reporters, consumers and physicians reported the most AEs in 44.70% and 40.20%, respectively. Over the full years, the number of adverse reactions reported varied slightly, ranging from 343 cases in 2021 to 517 cases in 2023. Extrapolating from the number of adverse events reported in the first quarter of 2024, the number of adverse events in 2024 is approximately the same as the average number of events reported in previous years.

**TABLE 1:** Demographic characteristics of patients with onasemnogene abeparvovec (Zolgensma)-related AEs and IMEs in FAERS.

Characteristic	AEs	IMEs
Total	1951	778
Sex		
Female	892 (45.7%)	377 (48.46%)
Male	781 (40.0%)	299 (38.43%)
Missing	278 (14.2%)	102 (13.11%)
Age		
0∼6 months	361 (18.50%)	149 (19.15%)
6∼18 months	486 (24.91%)	251 (32.26%)
1.5∼10 years	303 (15.53%)	162 (20.82%)
10∼18 years	4 (0.21%)	1 (0.13%)
18∼ years	1 (0.05%)	0 (0.00%)
Missing	796 (40.80%)	215 (27.63%)
Outcome		
Death	110 (5.64%)	100 (12.85%)
Life-threatening	48 (2.46%)	44 (5.66%)
Disability	3 (0.15%)	1 (0.13%)
Hospitalization	489 (25.06%)	289 (37.15%)
Other serious events	441 (22.60%)	277 (35.60%)
Missing information	860 (44.08%)	67 (8.61%)
Occupation of Reporter		
Consumer	872 (44.7%)	196 (25.19%)
Health Professional	248 (12.7%)	118 (15.17%)
Physician	784 (40.2%)	450 (57.84%)
Other healthcare professional	15 (0.8%)	4 (0.51%)
Pharmacist	20 (1.0%)	6 (0.77%)
Missing	12 (0.6%)	4 (0.51%)
Reporter’s country		
United States	1,090 (55.9%)	279 (35.86%)
Russian Federation	83 (4.3%)	44 (5.66%)
United Kingdom	63 (3.2%)	43 (5.53%)
Japan	60 (3.1%)	35 (4.5%)
Germany	50 (2.6%)	34 (4.37%)
Reporting year		
2019 (Q2-Q4)	96 (4.92%)	
2020	439 (25.27%)	
2021	343 (17.58%)	
2022	453 (23.22%)	
2023	517 (26.50%)	
2024 (Q1)	103 (5.28%)	

### 3.2 Onasemnogene abeparvovec-associated AEs

The signal intensities of onasemnogene abeparvovec-associated AEs categorized by System Organ Classes (SOCs) are demonstrated in Table 2. A total of 26 organ systems were affected by adverse events linked to onasemnogene abeparvovec, as indicated by our statistical analysis. Among these, several significant SOCs were identified based on meeting the criteria of at least one of the four indices utilized for analysis. The significant SOCs included gastrointestinal disorders [case = 1,045, ROR 1.42 (95%CI 1.33–1.52)]; investigations [case = 3,155, ROR 7.84 (95%CI 7.52–8.18)]; infections and infestations [case = 857, ROR 1.63 (95%CI 1.52–1.75)]; respiratory, thoracic and mediastinal disorders [case = 809, ROR 1.94 (95%CI 1.8–2.08)]; metabolism and nutrition disorders [case = 393 ROR 2.17 (95%CI 1.96–2.4)]; blood and lymphatic system disorders [case = 513, ROR 3.29 (95%CI 3.01–3.59)]; hepatobiliary disorders [case = 216 ROR 2.8 (95%CI 2.45–3.2)]; and congenital, familial and genetic disorders [case = 45 ROR 1.69 (95%CI 1.26–2.27)]. These results underscore the specific organ systems most frequently associated with onasemnogene abeparvovec-induced adverse events, emphasizing the need for further investigation and attention in these areas.

**TABLE 2:** Signal strength of reports of onasemnogene abeparvovec (Zolgensma) at the System Organ Class (SOC) level in FAERS database.

System organ class (SOC)	Case numbers	ROR (95% two-sided CI)	PRR (χ^2^)	EBGM (EBGM 05)	IC (IC 025)
Investigations	3,155	7.84 (7.52–8.18)	5.6 (12624.03)	5.59 (5.39)	2.48 (0.82)
General disorders and administration site conditions	1,069	0.59 (0.55–0.63)	0.63 (274.04)	0.63 (0.6)	−0.66 (−2.32)
Gastrointestinal disorders	1,045	1.42 (1.33–1.52)	1.38 (116.1)	1.37 (1.3)	0.46 (−1.21)
Infections and infestations	857	1.63 (1.52–1.75)	1.58 (192.3)	1.58 (1.49)	0.66 (−1.01)
Respiratory, thoracic and mediastinal disorders	809	1.94 (1.8–2.08)	1.86 (336.11)	1.86 (1.75)	0.89 (−0.77)
Blood and lymphatic system disorders	513	3.29 (3.01–3.59)	3.16 (771.18)	3.16 (2.93)	1.66 (−0.01)
Metabolism and nutrition disorders	393	2.17 (1.96–2.4)	2.12 (237.3)	2.12 (1.95)	1.08 (−0.58)
Nervous system disorders	307	0.42 (0.37–0.47)	0.44 (239.09)	0.44 (0.4)	−1.19 (−2.86)
Hepatobiliary disorders	216	2.8 (2.45–3.2)	2.76 (243.79)	2.76 (2.46)	1.46 (−0.2)
Cardiac disorders	209	1.12 (0.98–1.29)	1.12 (2.65)	1.12 (1)	0.16 (−1.51)
Renal and urinary disorders	181	0.94 (0.81–1.09)	0.94 (0.7)	0.94 (0.83)	−0.09 (−1.75)
Psychiatric disorders	170	0.32 (0.27–0.37)	0.33 (243.16)	0.33 (0.29)	−1.6 (−3.26)
Skin and subcutaneous tissue disorders	154	0.26 (0.23–0.31)	0.28 (310.22)	0.28 (0.24)	−1.86 (−3.52)
Musculoskeletal and connective tissue disorders	133	0.26 (0.22–0.31)	0.27 (278.27)	0.27 (0.23)	−1.89 (−3.56)
Vascular disorders	133	0.74 (0.62–0.88)	0.74 (11.94)	0.74 (0.64)	−0.43 (−2.09)
Injury, poisoning and procedural complications	109	0.08 (0.07–0.1)	0.09 (1093.9)	0.09 (0.08)	−3.42 (−5.09)
Congenital, familial and genetic disorders	45	1.69 (1.26–2.27)	1.69 (12.75)	1.69 (1.32)	0.76 (−0.91)
Eye disorders	37	0.2 (0.14–0.28)	0.2 (117.74)	0.2 (0.16)	−2.3 (−3.96)
Immune system disorders	29	0.25 (0.17–0.36)	0.25 (64.66)	0.25 (0.19)	−1.98 (−3.65)
Ear and labyrinth disorders	11	0.28 (0.15–0.5)	0.28 (20.91)	0.28 (0.17)	−1.85 (−3.52)
Endocrine disorders	10	0.38 (0.21–0.71)	0.38 (9.85)	0.39 (0.23)	−1.38 (−3.04)
Neoplasms benign, malignant and unspecified (incl cysts and polyps)	9	0.02 (0.01–0.05)	0.02 (356.02)	0.02 (0.01)	−5.32 (−6.99)
Product issues	8	0.04 (0.02–0.09)	0.05 (162.17)	0.05 (0.03)	−4.45 (−6.12)
Reproductive system and breast disorders	4	0.06 (0.02–0.17)	0.07 (53.97)	0.07 (0.03)	−3.94 (−5.61)
Surgical and medical procedures	3	0.02 (0.01–0.07)	0.02 (133.91)	0.02 (0.01)	−5.52 (−7.19)
Social circumstances	1	0.02 (0–0.16)	0.02 (43.26)	0.02 (0)	−5.49 (−7.16)

Notes: Red are those that follow the algorithm.

Onasemnogene abeparvovec-related adverse reaction reports were calculated using the ROR, PRR, BCPNN and MGPS methods to obtain 295 signals. Subsequently, 14 signals that were deemed unrelated to medication, such as symptoms of disease progression, various types of injuries, surgical complications, and medical manipulations, were excluded. This resulted in 281 signals involving 20 SOCs, detailed in Supplementary Table S2. Table 3 summarizes the reported preferred terms (PTs) that occurred at least 20 times, encompassing 50 PTs corresponding to 12 SOCs, covering 12 IMEs. More importantly, our data mining revealed several significant AEs that were not explicitly mentioned in the onasemnogene abeparvovec prescribing information. The unexpected AEs consist of PTs such as leukopenia, elevated C-reactive protein levels, tachycardia, bradycardia, salivary hypersecretion, hyperkalemia, among others. Our analysis has identified additional AEs that highlight and enhance the overall comprehension of onasemnogene abeparvovec’s safety profile.

**TABLE 3:** Signal strength of reports of onasemnogene abeparvovec (Zolgensma) at the PT level in the FAERS database.

SOC name	Preferred terms (PTs)	Case	ROR (95% two-sided CI)	PRR (χ^2^)	EBGM (EBGM05)	IC (IC025)	IMEs
Blood and lymphatic system disorders	Thrombocytopenia	201	12.66 (11.01–14.57)	12.42 (2103.23)	12.36 (10.99)	3.63 (1.96)	YES
	Thrombotic microangiopathy	39	25.4 (18.51–34.84)	25.3 (901.06)	25.05 (19.23)	4.65 (2.98)	YES
	Leukopenia	36	4.93 (3.55–6.84)	4.91 (112.09)	4.91 (3.73)	2.29 (0.63)	YES
Cardiac disorders	Tachycardia	63	5.13 (4–6.57)	5.1 (207.55)	5.09 (4.14)	2.35 (0.68)	NO
	Bradycardia	42	5.57 (4.11–7.55)	5.55 (156.58)	5.54 (4.3)	2.47 (0.8)	YES
Gastrointestinal disorders	Vomiting	488	8.14 (7.43–8.92)	7.78 (2893.84)	7.76 (7.19)	2.96 (1.29)	NO
	Salivary hypersecretion	26	19.23 (13.06–28.29)	19.18 (444.55)	19.04 (13.78)	4.25 (2.58)	NO
General disorders and administration site conditions	Pyrexia	572	11.82 (10.86–12.86)	11.17 (5302.04)	11.13 (10.36)	3.48 (1.81)	NO
Hepatobiliary disorders	Hepatic function abnormal	36	6.66 (4.8–9.25)	6.64 (172.12)	6.63 (5.04)	2.73 (1.06)	NO
	Hypertransaminasaemia	35	25.81 (18.48–36.02)	25.71 (822.87)	25.46 (19.26)	4.67 (3)	NO
Infections and infestations	Rhinovirus infection	50	65.94 (49.75–87.38)	65.6 (3098.1)	63.92 (50.5)	6 (4.33)	NO
	Respiratory syncytial virus infection	40	22.9 (16.76–31.28)	22.81 (826.53)	22.61 (17.41)	4.5 (2.83)	NO
	Respiratory tract infection	27	6.51 (4.46–9.51)	6.5 (125.35)	6.48 (4.73)	2.7 (1.03)	NO
	Upper respiratory tract infection	26	3.86 (2.63–5.68)	3.86 (54.93)	3.85 (2.79)	1.95 (0.28)	NO
	Viral infection	24	5.03 (3.37–7.52)	5.02 (77.23)	5.02 (3.59)	2.33 (0.66)	NO
Investigations	Aspartate aminotransferase increased	338	58.07 (52.03–64.8)	56.06 (17881.32)	54.83 (50.02)	5.78 (4.11)	NO
	Alanine aminotransferase increased	305	42.49 (37.88–47.67)	41.18 (11768.17)	40.51 (36.8)	5.34 (3.67)	NO
	Hepatic enzyme increased	245	23.22 (20.44–26.38)	22.66 (5030.74)	22.46 (20.19)	4.49 (2.82)	NO
	Platelet count decreased	166	9.75 (8.36–11.38)	9.6 (1276.66)	9.57 (8.41)	3.26 (1.59)	NO
	Troponin i increased	132	644.16 (531.38–780.86)	635.32 (66411.99)	504.9 (429.8)	8.98 (7.31)	NO
	Liver function test increased	123	29.29 (24.49–35.02)	28.93 (3278.91)	28.6 (24.62)	4.84 (3.17)	NO
	Transaminases increased	108	33.94 (28.04–41.08)	33.57 (3367.43)	33.13 (28.23)	5.05 (3.38)	NO
	Troponin increased	87	80.28 (64.79–99.49)	79.57 (6538.19)	77.1 (64.43)	6.27 (4.6)	NO
	Blood lactate dehydrogenase increased	85	50.39 (40.61–62.52)	49.95 (3996.94)	48.97 (40.89)	5.61 (3.95)	NO
	Oxygen saturation decreased	64	6.37 (4.98–8.15)	6.34 (287.3)	6.32 (5.15)	2.66 (0.99)	NO
	Body temperature increased	50	15.88 (12.01–20.98)	15.8 (688.95)	15.71 (12.44)	3.97 (2.31)	NO
	Troponin t increased	38	221.91 (159.13–309.46)	221.04 (7636.06)	202.86 (153.58)	7.66 (5.99)	NO
	Platelet count increased	37	16.77 (12.13–23.18)	16.7 (542.71)	16.6 (12.66)	4.05 (2.39)	NO
	Blood creatine phosphokinase increased	36	12.24 (8.82–16.99)	12.2 (368.33)	12.14 (9.23)	3.6 (1.94)	NO
	White blood cell count increased	36	7.55 (5.44–10.49)	7.53 (203.35)	7.51 (5.71)	2.91 (1.24)	NO
	Gamma-glutamyltransferase increased	35	14.32 (10.27–19.98)	14.28 (429.72)	14.2 (10.75)	3.83 (2.16)	NO
	Monocyte count increased	28	41.25 (28.38–59.96)	41.13 (1078.36)	40.47 (29.59)	5.34 (3.67)	NO
	Blood creatinine decreased	26	52.24 (35.4–77.07)	52.1 (1276.05)	51.04 (36.86)	5.67 (4.01)	NO
	Blood bilirubin increased	24	7.93 (5.31–11.84)	7.91 (144.42)	7.89 (5.64)	2.98 (1.31)	NO
	C-reactive protein increased	22	3.32 (2.18–5.04)	3.31 (35.51)	3.31 (2.33)	1.73 (0.06)	NO
Metabolism and nutrition disorders	Hyperkalaemia	21	4.63 (3.02–7.11)	4.62 (59.51)	4.61 (3.22)	2.21 (0.54)	YES
Nervous system disorders	Lethargy	32	4.65 (3.28–6.58)	4.63 (91.09)	4.63 (3.46)	2.21 (0.54)	NO
Psychiatric disorders	Irritability	63	10.34 (8.07–13.25)	10.28 (525.82)	10.24 (8.32)	3.36 (1.69)	NO
Renal and urinary disorders	Proteinuria	32	10.28 (7.26–14.55)	10.25 (265.94)	10.21 (7.63)	3.35 (1.68)	NO
	Haematuria	22	4.89 (3.22–7.44)	4.88 (67.86)	4.88 (3.44)	2.29 (0.62)	NO
Respiratory, thoracic and mediastinal disorders	Respiratory distress	48	14.33 (10.78–19.04)	14.26 (588.59)	14.18 (11.18)	3.83 (2.16)	YES
	Rhinorrhoea	46	4.36 (3.26–5.82)	4.34 (118.13)	4.33 (3.4)	2.12 (0.45)	NO
	Respiratory failure	43	4.7 (3.48–6.34)	4.68 (124.27)	4.67 (3.64)	2.22 (0.56)	YES
	Respiratory disorder	42	9.83 (7.26–13.32)	9.79 (330.4)	9.76 (7.57)	3.29 (1.62)	NO
	Atelectasis	38	37.19 (26.98–51.26)	37.04 (1313.03)	36.51 (27.91)	5.19 (3.52)	NO
	Increased bronchial secretion	25	79.39 (53.28–118.29)	79.18 (1869.63)	76.74 (54.97)	6.26 (4.59)	NO
	Aspiration	24	17.99 (12.03–26.89)	17.94 (381.27)	17.82 (12.73)	4.16 (2.49)	YES
	Acute respiratory failure	23	7.59 (5.04–11.43)	7.57 (130.79)	7.55 (5.36)	2.92 (1.25)	YES
	Tachypnoea	22	10.8 (7.1–16.43)	10.78 (194.41)	10.74 (7.56)	3.42 (1.76)	NO
	Hypoxia	22	4.2 (2.76–6.39)	4.19 (53.45)	4.19 (2.95)	2.07 (0.4)	YES

We then conducted subgroup analyses, which can reduce the confounding of the results by demographic characteristics to some extent (de Vries et al., 2020; Zou et al., 2024). Among the three subgroups aged 0–6 months, 6–18 months, and >18 months, the number of adverse reactions over 18 months (125 cases) was much higher than in the other two groups, almost twice as many as those reported under 6 months (69 cases). Additionally, when analysing the reported AEs with at least 10 occurrences in each subgroup, we found that signals reported only under 6 months included “decreased appetite,” “irritability,” “thrombocytosis,” while “leukopenia,” “salivary hypersecretion,” “increased bronchial secretion,” “blood creatine phosphokinase mb increased,” and “haematuria” were the only ones not reported within 6 months in the subgroup. On the other hand, “nausea,” “respiratory disorder,” “pneumonia aspiration,” “muscular weakness,” “parainfluenzae virus infection,” and “hepatomegaly” appeared to be more frequent in the 6–18 months subgroup, but we did not detect a signal for “thrombotic microangiopathy” in this group. In contrast, exclusive AEs in the subgroup aged >18 months subgroup included “oxygen saturation decreased,” “blood creatinine increased,” “respiratory distress,” “respiratory syncytial virus infection,” “tachycardia,” “rhinovirus infection,” “heart rate increased,” and “troponin t increased,” while “white blood cell count decreased” did not appear in this group (Supplementary Figure S2).

Similarly, subgroup analyses were conducted to examine and contrast similarities and variances in signals among different subgroups based on sex (Supplementary Figure S3) and reported person (Supplementary Figure S4). This data is crucial for enhancing clinical management practices, assisting clinical decision-makers in tailoring treatments according to the unique characteristics of specific subgroups.

### 3.3 Time to onset of onasemnogene abeparvovec-associated adverse events

**FIGURE 1:**
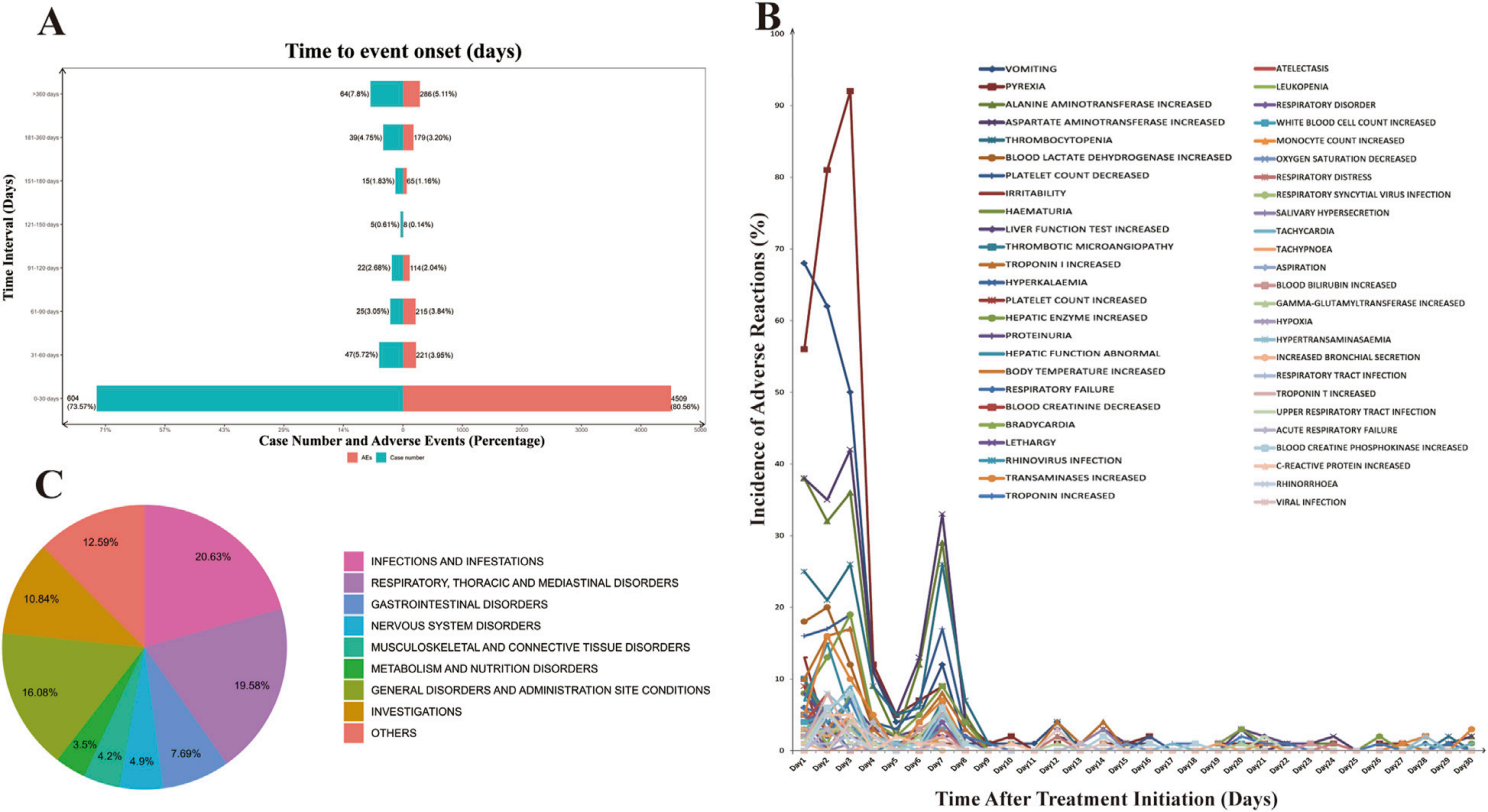
Time to Onset of Adverse Events Associated with Onasemnogene Abeparvovec (Zolgensma). **(A)** Comprehensive Analysis of the Temporal Onset of Adverse Events Associated with Onasemnogene Abeparvovec (Zolgensma). The incidence of adverse events and the number of patients affected exhibit a peak within the first month following Zolgensma administration. Subsequently, there is a marked decrease, with the lowest point occurring between days 120 and 150 post-administration, after which there is a gradual increase in the number of reported cases. **(B)** Incidence of Adverse Reactions in the First Month of Onasemnogene Abeparvovec (Zolgensma) Therapy. Adverse reactions reach their peak within the initial 2–3 days post-Zolgensma administration, followed by a precipitous decline. A minor resurgence is noted on day 7, beyond which the rate of adverse reactions plateaus. **(C)** Pie Chart of Morbidity Statistics After One Year of Onasemnogene Abeparvovec (Zolgensma) Treatment by System Organ Class (SOC).

The database provided contains information pertaining to the temporal initiation of negative occurrences linked to onasemnogene abeparvovec. Among the documented adverse events, a total of 5,597 cases were reported, with comprehensive and precise details regarding the time of occurrence. Analysis of the distribution of adverse event onset times, as depicted in Figure 1A, reveals a peak in patient count and frequency of adverse events within the initial month. Subsequently, there is a progressive decline in occurrences in the following months, reaching a nadir in the fifth month with isolated instances, succeeded by a gradual rise in adverse reactions.

Subsequently, we conducted a detailed analysis of the incidence of adverse reactions during the initial month of onasemnogene abeparvovec treatment (Figure 1B). During the initial three-day period of treatment, adverse reactions were observed with a high frequency, accounting for 50.54% of all reported adverse reactions. This was followed by a slight decline, with a subsequent increase on the seventh day, reaching a second peak. At this juncture, the adverse reactions were predominantly classified as “aspartate aminotransferase increased” and “alanine aminotransferase increased,” “thrombocytopenia,” “platelet count decreased,” and “vomiting.” These observations suggested an activation of the body’s immune response to the treatment. Following this, there was a swift decline in adverse reactions, leading to a stabilized level. The results demonstrated an elevation in both transferase and alanine aminotransferase levels, accompanied by a reduction in platelet and haemoglobin counts. Additionally, the patient displayed symptoms of vomiting. These findings indicate that the patient’s immune system was activated, resulting in a rapid decline in these parameters and a subsequent stabilisation. One year later, the most commonly reported adverse reactions were infections and infestations, respiratory system, gastrointestinal system, nervous system, musculoskeletal and connective tissue, and metabolic systems, accounting for more than 60% of the total adverse reactions. These findings emphasize the importance of monitoring potential AEs in patients throughout the course of onasemnogene abeparvovec therapy, even beyond the initial year (Figure 1C).

### 3.4 Sex differences in risk signals for onasemnogene abeparvovec

**FIGURE 2:**
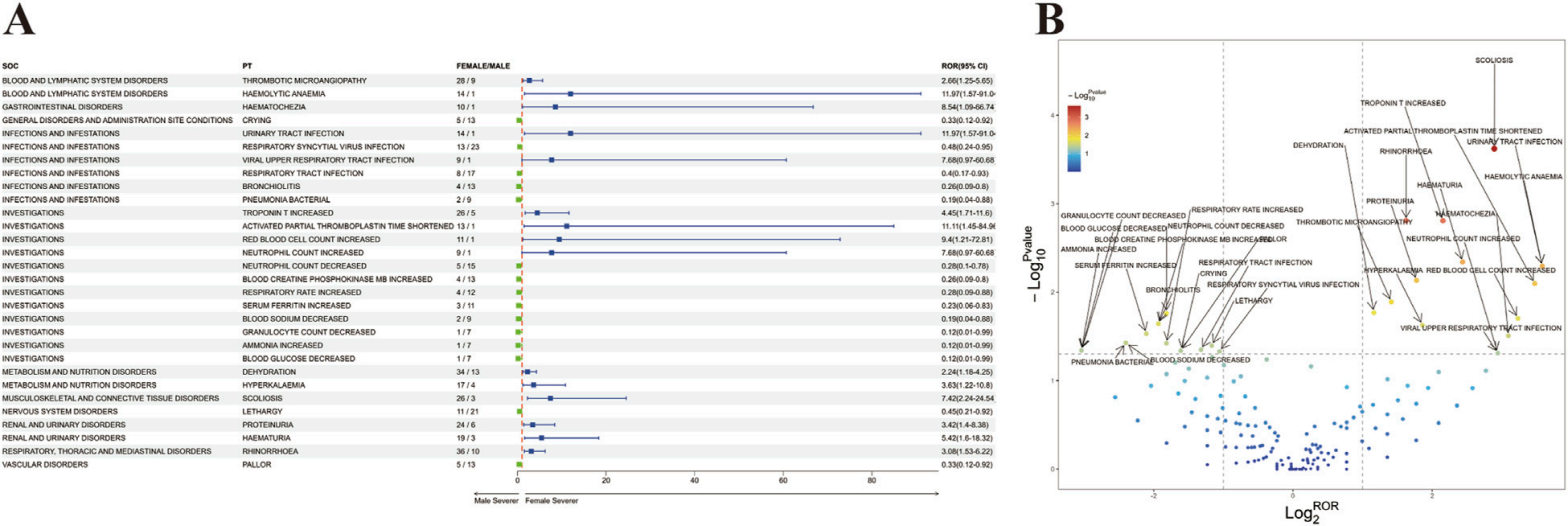
Analysis of Sex-Stratified Risk Signals for Onasemnogene Abeparvovec (Zolgensma). **(A)** Forest Plot of Sex-Associated Adverse Events (AEs). All preferred terms (PTs) correlated with sex were identified and are presented with their respective reporting odds ratios (RORs) and 95% confidence intervals (CIs) in a forest plot format. The color coding indicates the severity: blue denotes PTs with greater severity in females versus males, and green denotes PTs with greater severity in males versus females. **(B)** Volcano Plot of Sex-Stratified Risk Signals for Onasemnogene Abeparvovec (Zolgensma). The plot’s horizontal axis depicts the log2-transformed reporting odds ratios (log_2_^ROR^), while the vertical axis represents the negative logarithm (base 10) of the p-values (-log_10_^p-values^). Notable signals are distinctly highlighted and annotated with color differentiation. Abbreviations: PTs, preferred terms; SOC, System Organ Class; CI, confidence interval; ROR, reporting odds ratio.

In order to investigate the potential impact of sex on adverse reactions to onasemnogene abeparvovec, an analysis was conducted on preferred terms (PTs) exhibiting disproportionate incidence of adverse drug events (ADEs) between males and females. The study identified 67 unique PT reactions specific to females and 97 unique PT reactions specific to males. Additionally, adverse reactions present in both sexes were further examined using the Reporting Odds Ratio (ROR) method to identify PTs with a significance level of p < 0.05, categorized by SOC. The findings are illustrated in Figure 2A, with detailed results provided in Supplementary Table S3. Several AEs, such as crying, respiratory syncytial virus infection, respiratory tract infection, bronchiolitis, pneumonia bacterial, decreased neutrophil count, increased blood creatine phosphokinase MB, elevated respiratory rate, increased serum ferritin, decreased blood sodium, reduced granulocyte count, elevated ammonia, decreased blood glucose, lethargy, and pallor, were more frequently observed in males. Conversely, high-risk AEs noted in females included viral upper respiratory tract infection, increased troponin T, shortened activated partial thromboplastin time, elevated red blood cell count, increased neutrophil count, dehydration, hyperkalemia, scoliosis, proteinuria, hematuria, and rhinorrhea.

Furthermore, a “volcano diagram” was created to visually represent the signals and analyze the outcomes of sex-based variations in AE signal extraction for onasemnogene abeparvovec (Figure 2B), with each point denoting a onasemnogene abeparvovec-associated AE, and statistically significant points being specifically identified.

### 3.5 Analysis of concomitant medications with onasemnogene abeparvovec

**FIGURE 3:**
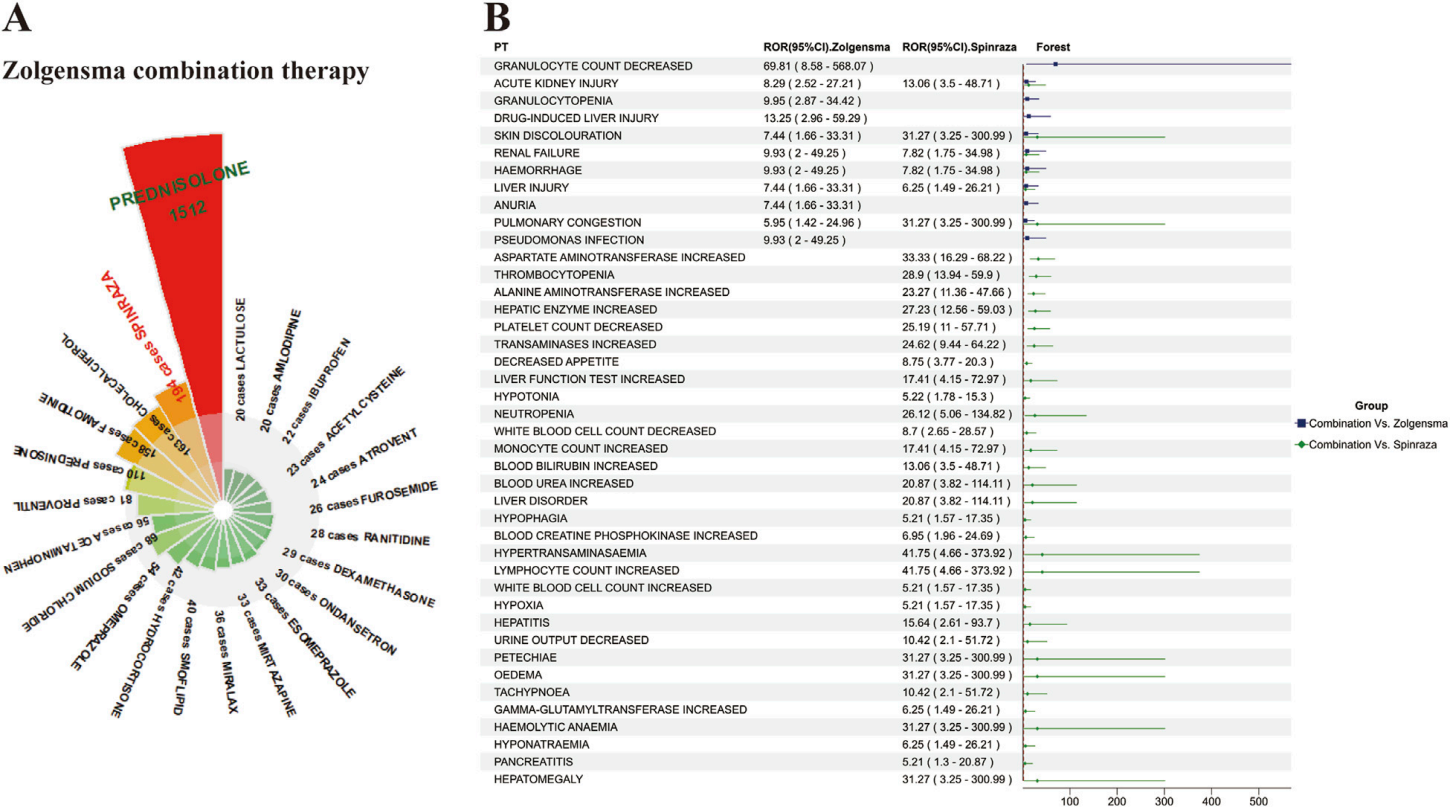
Analysis of Concomitant Medication Therapies with Onasemnogene Abeparvovec (Zolgensma) **(A)** Comprehensive Overview of Concomitant Medications with Onasemnogene Abeparvovec (Zolgensma). This figure details the frequency of co-administered drugs with Zolgensma, noting instances where the combination occurred in over 20 cases. Prednisolone is identified as the most commonly co-administered medication with Zolgensma, succeeded by Nusinersen (Spinraza), cholecalciferol, and famotidine. **(B)** Assessment of Adverse Reactions in the Combined Therapy of Onasemnogene Abeparvovec (Zolgensma) and Nusinersen (Spinraza). The color scheme utilized within this analysis denotes significant differences in adverse reactions when the drugs are co-administered versus Zolgensma monotherapy, with blue indicating a higher incidence in the combination group compared to Zolgensma alone, and green indicating an increased frequency in the combination therapy compared to Spinraza monotherapy. Abbreviations: PTs, preferred terms; CI, confidence interval; ROR, reporting odds ratio.

We counted 3,951 records of concomitant medications in the FAERS database in combination with onasemnogene abeparvovec, including glucocorticoids, histamine-2 blockers, bronchodilators, 5-HT3 receptor antagonists, and proton pump inhibitors, which serve to mitigate inflammation, regulate the hyperactive immune response, and address other physiological conditions. Interestingly, we found 194 cases of onasemnogene abeparvovec in combination with Spinraza, another drug for SMA (Figure 3A). Subsequently, we analysed these two drugs in combination, screening results that satisfied a minimum of three screening criteria for adverse drug interaction assessment. The combination of two drugs increased the adverse effects in patients compared to the single drug, with the increase in adverse events compared to onasemnogene abeparvovec alone being only 1/3 of the increase in cases with Spinraza alone. Among these, acute kidney injury, skin discolouration, renal failure. Haemorrhage, liver injury, and pulmonary congestion, all of which are significantly increased when used in combination, necessitating heightened vigilance (Figure 3B).

## 4 Discussion

Given the limited availability of clinical cases and preclinical evidence, it is imperative to gather pharmacovigilance information from post-market surveillance systems that report adverse events, as this can significantly enhance drug specifications. Previous research on onasemnogene abeparvovec has predominantly concentrated on its mechanism of action, clinical applications, safety assessment, and the effectiveness of combination therapies, with minimal focus on recent real-world investigations. Leveraging the most extensive dataset of real-world data, we have conducted an assessment of post-market drug surveillance for onasemnogene abeparvovec. The objective is to scrutinize novel and significant adverse reactions, facilitate the refinement and consolidation of product characteristics, and establish a foundation for informed clinical prescribing practices.

As indicated in our report (Table 1), the safety reports pertaining to onasemnogene abeparvovec exhibited a fluctuating trend, with the exception of the initial launch in 2019. This differs from the trends derived from the recent European Medicines Agency’s (EMA) pharmacovigilance database (Ruggiero et al., 2024) and previous drug comparative analyses (Zhuang et al., 2023). This discrepancy may be attributed to the utilization of distinct databases across different geographical regions. With regard to sex distribution, the incidence of common adverse reactions is marginally higher in females than in males, whlie in IMEs, the prevalence of these reactions is significantly higher in females than in males. This finding is consistent with the results of the European database analyses (Ruggiero et al., 2024). It is notable that consumers reported the highest number of AEs in our report, thanks to the transparency of public databases (Scavone et al., 2018; Ruggiero et al., 2024; Rossi et al., 2022). However, the primary source of serious adverse reactions remains physicians. Analysis of patient age revealed that the majority of reports, approximately four-fifths, pertained to patients under 2 years old, excluding cases with unknown age data (results not shown), which is in line with the emphasis on the infant population in clinical trial data.

Regarding the types of adverse reactions, our analysis findings reveal notable variations in the occurrence of organ-related adverse reactions such as investigations, blood and lymphatic system disorders, hepatobiliary disorders, metabolism and nutrition disorders (Table 2). The related adverse reactions are consistent with those observed in previous clinical studies, mainly manifested as fever, vomiting, elevated transaminases, thrombocytopenia, decreased platelet count, and elevated creatine kinase levels (Mendell et al., 2021; Strauss et al., 2022b; Chiriboga et al., 2023; Finkel et al., 2023; Gowda et al., 2024; Servais et al., 2024; Mendonça et al., 2024). It is worth noting that our statistical analysis highlights a considerable number of adverse reactions associated with the respiratory system and infections, such as rhinitis, nasal viral infections, respiratory ailments, and respiratory syncytial virus infections (Table 3).

Based to clinical adverse events and laboratory findings, liver function-related adverse events are primarily concentrated during the initial 8 days, followed by a smaller peak on the 14th and 20th days (Supplementary Figure S5), which is slightly different from the reported cases in clinical cases where liver-related adverse events such as aspartate aminotransferase (AST), alanine aminotransferase (ALT), and liver enzymes were observed to reach their first peak during the first week or so, followed by a second peak during weeks 3–6 (Day et al., 2021b; Matesanz et al., 2021; D’Silva et al., 2022; Bitetti et al., 2023; Stettner et al., 2023; Mendonça et al., 2024). Nevertheless, this is consistent with the timing of dosing in German teams and dosing in cynomolgus monkeys (Hudry et al., 2023), indicating individual differences in liver injury-related reactions among different patients. Lee et al. (2022) corroborated the existence of notable discrepancies in the peaks and durations of AST and ALT by conducting a follow-up analysis of the short-term clinical outcomes of five cases. A meta-analysis of 250 patients (Yang et al., 2023) also demonstrated that the prevalence of transaminase elevations differed significantly between different age groups. Our data underscore the importance of monitoring the initial 3 days post-administration, particularly the second day, when severe adverse reactions like acute hepatic failure, gallbladder enlargement, and ischemic hepatitis are most concentrated. The hepatotoxicity mechanism of onasemnogene abeparvovec is thought to be driven by immune reactions in the liver triggered by viral transduction and transgene expression. Hepatic injury typically manifasts concurrently with the emergence of anti-AAV9 antibodies, as evidenced in animal models (Bethesda, 2012; Chand et al., 2021a; Hudry et al., 2023) and in analogous adverse effects observed in other AAV drug-treated diseases (Manno et al., 2006; Kattenhorn et al., 2016; Colella et al., 2018; Chand et al., 2021a; Hamilton and Wright, 2021; Maestro et al., 2021), suggesting the necessity for replacing gene expression driven by the strong cytomegalovirus enhancer/chicken beta-actin (CMVen/CB) promoter with tissue-specific promoters to restore or approach the physiological levels of SMN expression, as demonstrated by Xie et al. (2024). In addition, it is noteworthy that the clinical treatment of SMA patients with onasemnogene abeparvovec often necessitates the use of concomitant medications to suppress comorbidities. Chand et al. (2021a) analysed 325 SMA patients and found that over 40% of patients received concomitant medications that may have hepatotoxicity, with common medications being acetaminophen, ibuprofen, and ranitidine, which align with the most common concomitant medications identified in our analysis (Figure 3A). It is recommended that, where possible, the administration of potentially hepatotoxic drugs should be avoided for a minimum of 1 week prior to and following the administration of onasemnogene abeparvovec, given that the majority of liver-related adverse reactions are reported within 1 week of administration (Chand et al., 2021a).

Case reports have identified elevated cardiac troponin levels as indicative of a cardiac event (Mendell et al., 2021; Lee et al., 2022; NPS MedicineWise, 2022; Weiß et al., 2022; Chencheri et al., 2023; Chiriboga et al., 2023). Preclinical mouse studies have shown that the heart may be a target organ for toxicity, as evidenced by intracardiac thrombosis (Chand et al., 2023). In our data, common adverse events following onasemnogene abeparvovec treatment include elevated Troponin I and/or Troponin T levels, as well as tachycardia and bradycardia (Table 3; Supplementary Table S2). However, there was no clinicopathological evidence of intracardiac thrombosis or cardiomyopathy, cardiac failure, and so forth. From the available clinical reports, most of the abnormal cardiac troponin levels following onasemnogene abeparvovec treatment are accompanied by transaminases, particularly alanine aminotransferase (ALT) and aspartate aminotransferase (AST), in the setting of liver injury or hepatitis. Some research suggest that elevated cardiac troponin levels are common in patients with acute and subacute liver failure, implying secondary myocardial injury in cases of multi-organ involvement under metabolic stress (Parekh et al., 2007; Jaber and Paugam-Burtz, 2013; Chand et al., 2023). Finally, adenovirus can cause myocarditis, and while it cannot be ruled out that this vector may induce mild transient cardiac inflammation in a small subset of patients, the available human data in patients receiving onasemnogene abeparvovec treatment do not support this hypothesis (Bejiqi et al., 2019). A clinical study found significant age-related variations in high-sensitive cardiac troponin I (hs-cTnI) concentrations in newborns, emphasizing the necessity of pre-treatment measurements in interpreting changes in hs-cTnI values post-treatment, as these changes may more significantly indicate treatment-related myocardial injury compared to baseline values (Johannsen et al., 2023). In conclusion, cardiac troponin monitoring is recommended in product labelling following onasemnogene abeparvovec administration based on cardiac findings documented in mice. The translatability of rodent study results to humans, as questioned by non-human primate toxicology studies and available clinical data (Day et al., 2021b), does not support the assumption of direct and significant cardiac toxicity of onasemnogene abeparvovec in humans. SMA may be associated with cardiac events arising from underlying diseases and secondary complications. Healthcare professionals should apply medical judgement in assessing the etiology and evaluation of cardiac events following onasemnogene abeparvovec administration, considering all possibilities and managing patients accordingly (Chand et al., 2023).

**FIGURE 4:**
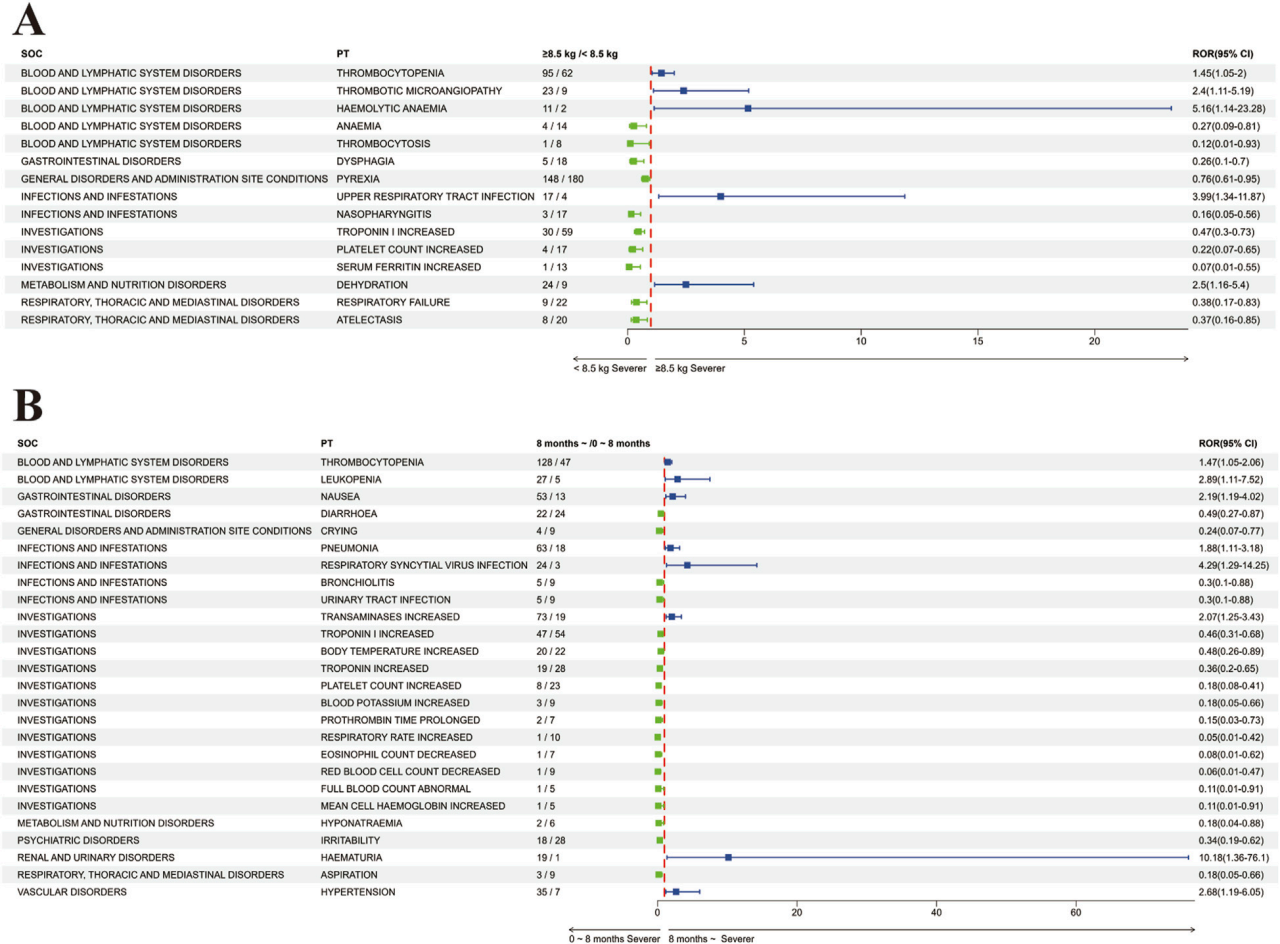
Comparative Analysis of Adverse Reactions in Subgroups Post-Onasemnogene Abeparvovec (Zolgensma) Administration **(A)** Forest Plot Assessing Adverse Reactions Based on Weight Category Following Onasemnogene Abeparvovec (Zolgensma) Administration. This plot compares adverse reactions in patients categorized by a weight threshold of 8.5 kg. Preferred terms (PTs) with increased severity in patients weighing 8.5 kg or more are denoted in blue, whereas those with increased severity in patients weighing less than 8.5 kg are indicated in green. **(B)** Forest Plot Evaluating Adverse Reactions Based on Age Group Following Onasemnogene Abeparvovec (Zolgensma) Administration. This analysis examines adverse reactions in infants stratified by age, above and below 8 months. Preferred terms (PTs) with greater severity in patients older than 8 months are represented in blue, and those with greater severity in patients younger than 8 months are shown in green. Abbreviations: SOC, System Organ Class; PT, Preferred Term; ROR (95%CI), Reporting Odds Ratio with 95% Confidence Interval.

Common adverse reactions also include thrombocytopenia, platelet count decreased, and thrombotic microangiopathy. In our data, reports of decreased platelets rank second only to fever, vomiting, and increased liver enzymes (Table 3; Supplementary Table S2). Previous studies have shown discrepancies in the occurrence of platelet decrease after onasemnogene abeparvovec treatment in different developmental stages and weight categories. A Brazilian cohort study (Mendonça et al., 2024) found an association between platelet decrease and weight increase, while Servais et al. (2024) found that the incidence of transient platelet decrease during treatment was similar in patients weighing <8.5 kg and ≥8.5 kg. Our study found that platelet decrease is more common in patients weighing over 8.5 kg [ROR 1.45 (95%CI 1.05–2)] (Figure 4A), possibly due to a smaller sample size in Laurent Servais’ data (<8.5 kg Vs. ≥8.5 kg = 120 Vs. 21), leading to significant bias in the statistical results. Thrombotic microangiopathy is a rare, acute, life-threatening disease characterized by platelet decrease and microangiopathic hemolytic anemia, with platelet decrease being the main feature. We identified a total of 39 cases of thrombotic microangiopathy, which were also more common in patients weighing over 8.5 kg (Figure 4A). Additionally, our analysis showed that the probability of platelet decrease and elevated transaminases occurring in patients over 8 months of age was significantly higher than in patients under 8 months of age (Figure 4B), consistent with the study by Yang et al. (2023). Similarly, we also found a higher number of reports of adverse reactions related to kidney damage such as proteinuria, haematuria, blood creatinine decreased, and hyperkalaemia (Table 3), which are often associated with the occurrence of TMA (Chand et al., 2021a; Blair, 2022; Guillou et al., 2022; Witte et al., 2022; Yazaki et al., 2022; Mendonça et al., 2024), and TMA can lead to renal failure (Nigro et al., 2023), therefore, if TMA is clinically suspected, further hemoglobin testing, as well as tests for hemolysis and renal dysfunction (Day et al., 2021b; Yang et al., 2023) are also needed. Additionally, the Phase III SPR1NT trial (Strauss et al., 2022a) also observed cases of areflexia and hyporeflexia, both of which are part of the standard for radiculoplexus neuropathy-related AEs; however, all symptoms were mild and considered unrelated to treatment. In our analysis, areflexia (n = 7) and hyporeflexia (n = 2) also appeared in adverse drug reactions (ADRs) (Supplementary Table S2). Nevertheless, in non-human primates, intrathecal administration has been observed to cause radiculoplexus neurotoxicity (Hinderer et al., 2018; Hordeaux et al., 2020; Tukov et al., 2022; Hudry et al., 2023; Perera et al., 2024), indicating the importance of monitoring it as a potential adverse event (Hjartarson et al., 2022). Immune-mediated adverse events, including those mentioned, may necessitate hospitalization and intravenous administration of steroids and other immunosuppressants post-treatment.

It is worth noting that we have found respiratory system adverse events (respiratory failure, respiratory distress, increased bronchial secretions, etc.) and respiratory tract infections (respiratory syncytial virus infection, respiratory tract infection, viral infection, rhinovirus infection) also require special attention (Table 3), which is consistent with previous clinical research reports (Finkel et al., 2014; Finkel et al., 2023; Kolb et al., 2017; Hoy, 2019; Day et al., 2021a; Mendell et al., 2021; Mercuri et al., 2021; Erdos and Wild, 2022; McMillan et al., 2022), and respiratory complications are the leading cause of morbidity and mortality in infants severely affected by SMA (D’Silva et al., 2022), although researchers of the STR1VE study believe that these respiratory adverse events are due to the potential SMA disease process (Mendell et al., 2021; Blair, 2022), unrelated to onasemnogene abeparvovec treatment (Mendell et al., 2021; Strauss et al., 2022b; Strauss et al., 2022a). However, in mouse experiments, supraphysiological expression of SMN in the skeletal muscles and hearts of SMA mice receiving the standard vector may lead to cardiac and respiratory dysfunction (Xie et al., 2024). Active infection is a potential risk factor for developing thrombotic microangiopathy (Chand et al., 2021b), and also, corticosteroid therapy and potential adrenal insufficiency may exacerbate respiratory infections in SMA patients (Day et al., 2021a). This suggests the need to establish respiratory care to monitor cough, airway clearance, and potential hypoventilation (Kichula et al., 2021).

We identified several potential safety signals for the use of onasemnogene abeparvovec using the FAERS database that effectively overcome the constraints of limited sample sizes and brief observation periods in clinical trials. However, we could not completely rule out the possibility of the adverse events occurring due to the indications for the use of the drug rather than the drug itself. It is also important to consider the inherent limitations of the FAERS database (Chouchana et al., 2021), adverse event reports are voluntary and sourced from various channels, leading to potential underreporting, delayed reporting, and incomplete information that may introduce bias in the analysis of disproportionate reporting. Furthermore, even when the reports are complete, it is seldom possible to enumerate the denominator or potential user population, which precludes the calculation of incidence and risk (Crisafulli et al., 2023). Finally, while the disproportionality method can identify statistical correlations between a drug and adverse reactions, it does not establish causality (Xia et al., 2023). In light of the aforementioned shortcomings and other potential confounding factors and biases, it is imperative to exercise caution in interpreting the results of these analyses and to conduct further clinical studies to confirm these associations. Despite the constraints of the FAERS database in pharmacovigilance studies, our thorough analysis of the adverse event signals associated with onasemnogene abeparvovec and the discovery of previously unrecognized adverse event signals could serve as a foundation for future clinical research on this medication.

## 5 Conclusion

Our research aims to utilize the FAERS database to mine and analyse adverse reaction signals of onasemnogene abeparvovec in clinical practice, defining in the safety profile of the drug, with a view to providing some reference to improve the safety of clinical medication. Our research confirms that the first 8 days after administration are the peak period for the outbreak of adverse reactions and require thorough monitoring of the patient’s physical condition. Hepatotoxicity and thrombocytopenia are the main issues that occur in the clinical use of onasemnogene abeparvovec, which may lead to symptoms such as acute liver failure, ischemic hepatitis, and thrombotic microangiopathy. Thrombocytopenia and thrombotic microangiopathy are more prevalent in patients weighing ≥8.5 kg, and if thrombotic microangiopathy occurs, close attention to changes in kidney function is needed. Although the therapeutic relevance of cardiac adverse reactions to onasemnogene abeparvovec cannot be determined, cardiac monitoring is also more important given the potential for adverse reactions. For potential safety hazards, respiratory and dorsal root ganglion (DRG) toxicity warrant attention, especially for ongoing monitoring and long-term follow-up of the respiratory system and its complications.

## Data Availability

Publicly available datasets were analyzed in this study. This data can be found here: https://fis.fda.gov/extensions/FPD-QDE-FAERS/FPD-QDE-FAERS.html.
