# Supplementary material for: Comprehensive analysis of adverse events associated with onasemnogene abeparvovec (Zolgensma) in spinal muscular atrophy patients: insights from FAERS database
Source: Front Pharmacol. 2025 Jan 7;15:1475884. doi: 10.3389/fphar.2024.1475884 (PMC11747325; doi:10.3389/fphar.2024.1475884)
Supplement: Supplementary file 3 [file Table1.docx]

**Supplementary Table S1. Overview of Four Signal Detection Algorithms**

| Algorithms | Equation | Criteria |
| --- | --- | --- |
| ROR | ROR=ad/b/c | lower limit of 95% CI>1, N≥3 |
|  | 95%CI=e^ln(ROR)±1.96(1/a+1/b+1/c+1/d)^0.5^ |  |
| PRR | PRR=a(c+d)/c/(a+b) | PRR≥2, χ^2^≥4, N≥3 |
|  | χ^2^=[(ad-bc)^2](a+b+c+d)/[(a+b)(c+d)(a+c)(b+d)] |  |
| BCPNN | IC=log_2_a(a+b+c+d)(a+c)(a+b) | IC025>0 |
|  | 95%CI= E(IC) ± 2V(IC)^0.5 |  |
| MGPS | EBGM=a(a+b+c+d)/(a+c)/(a+b) | EBGM05>2 |
|  | 95%CI=e^ln(EBGM)±1.96(1/a+1/b+1/c+1/d)^0.5^ |  |

**Equation**: **a**, Number of reports for the target drug involving the target adverse reaction. **b**, Number of reports for the target drug involving other adverse reactions. **c**, Number of reports for other drugs involving the target adverse reaction. **d**, Number of reports for other drugs involving other adverse reactions. **95% CI**, 95% Confidence Interval, an estimate of the range within which the true population parameter is expected to lie with 95% confidence. **N**, The number of reports. **χ2**, Chi-squared statistic, a measure of the difference between observed and expected values in categorical data. **IC**, Information Component, a Bayesian metric used in the Bayesian Confidence Propagation Neural Network (BCPNN) algorithm. **IC025**, The lower limit of the 95% Confidence Interval for the Information Component. **E(IC)**, The expected value of the Information Component. **V(IC)**, The variance of the Information Component. **EBGM**, Empirical Bayesian Geometric Mean, a statistical measure used in the Multi-Item Gamma Poisson Shrinker (MGPS) algorithm. **EBGM05**, The lower limit of the 95% Confidence Interval for the Empirical Bayesian Geometric Mean.
