# Supplementary material for: Comprehensive analysis of adverse events associated with onasemnogene abeparvovec (Zolgensma) in spinal muscular atrophy patients: insights from FAERS database
Source: Front Pharmacol. 2025 Jan 7;15:1475884. doi: 10.3389/fphar.2024.1475884 (PMC11747325; doi:10.3389/fphar.2024.1475884)
Supplement: Supplementary file 5 [file DataSheet1.docx]

Supplementary Material


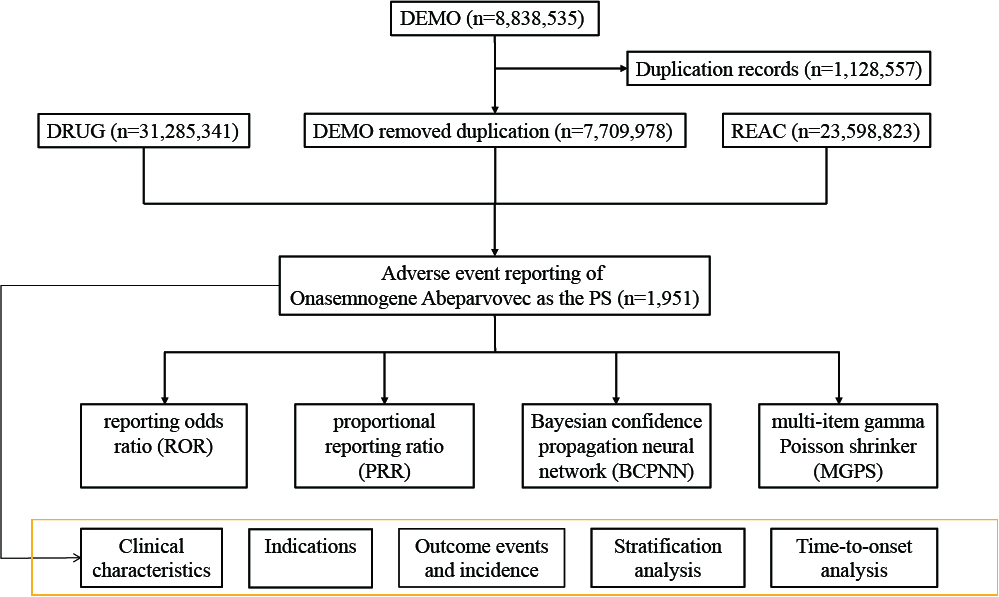


**Supplementary Figure S1. The flow chart of screening process for ZOLGENSMA-Associated Adverse Events from FAERS database.**

DEMO, demographic and administrative information; DRUG, drug information; REAC, adverse drug reaction information; PS, primary suspect drug.


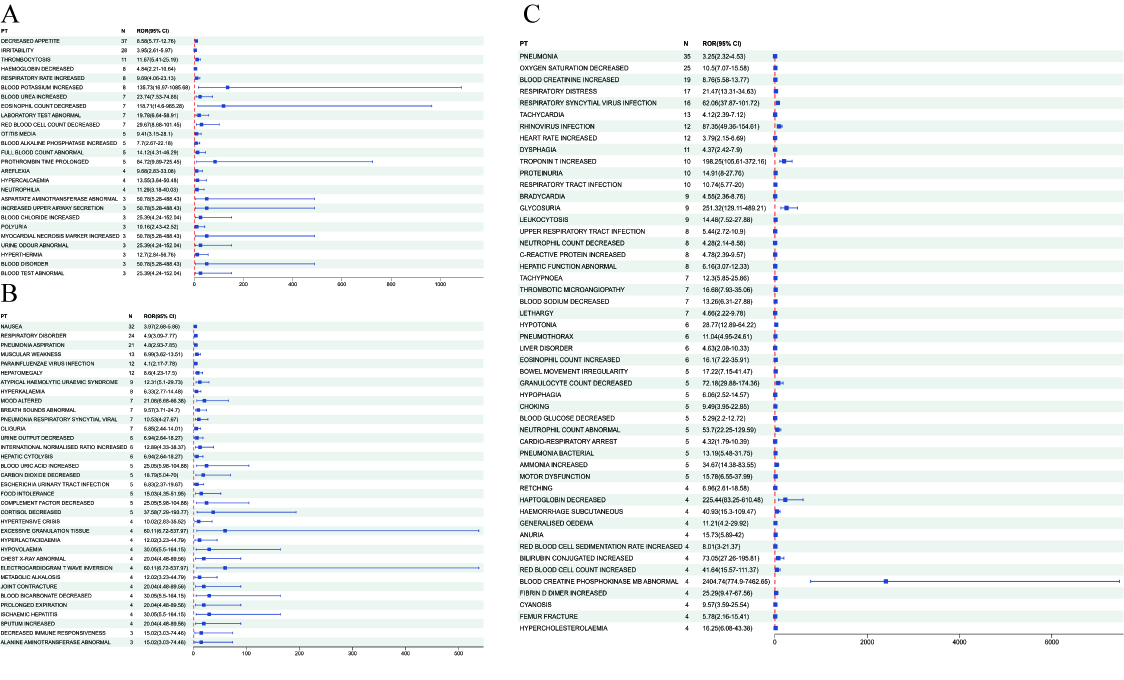


**Supplementary Figure S2. Age Subgroup Analysis of Adverse Reactions Associated with Onasemnogene Abeparvovec (Zolgensma).**

1. Forest Plot of Adverse Events Specific to Patients Aged 0-6 Months.
2. Forest Plot of Adverse Events Specific to Patients Aged 6-18 Months.
3. Forest Plot of Adverse Events Specific to Patients Over 18 Months of Age.

Analyses were conducted using age subgroups to identify adverse reactions that met the criteria of all four algorithms. Subsequent comparative analyses within each subgroup were performed to select adverse reactions specific to each subgroup and to screen for Preferred Terms (PTs) with a significance level of P<0.01 for presentation. If the number of subgroup-specific adverse reactions exceeded 50, the top 50 PTs were selected for reporting.

A total of 26 specific adverse events were identified for the 0-6 month subgroup, 35 for the 6-18 month subgroup, and 89 for the subgroup over 18 months of age.

**Abbreviations**: PT, Preferred Term; N, Number of Reports of Adverse Drug Reactions; ROR(95%CI), Reporting Odds Ratio (ROR) with a 95% Confidence Interval.


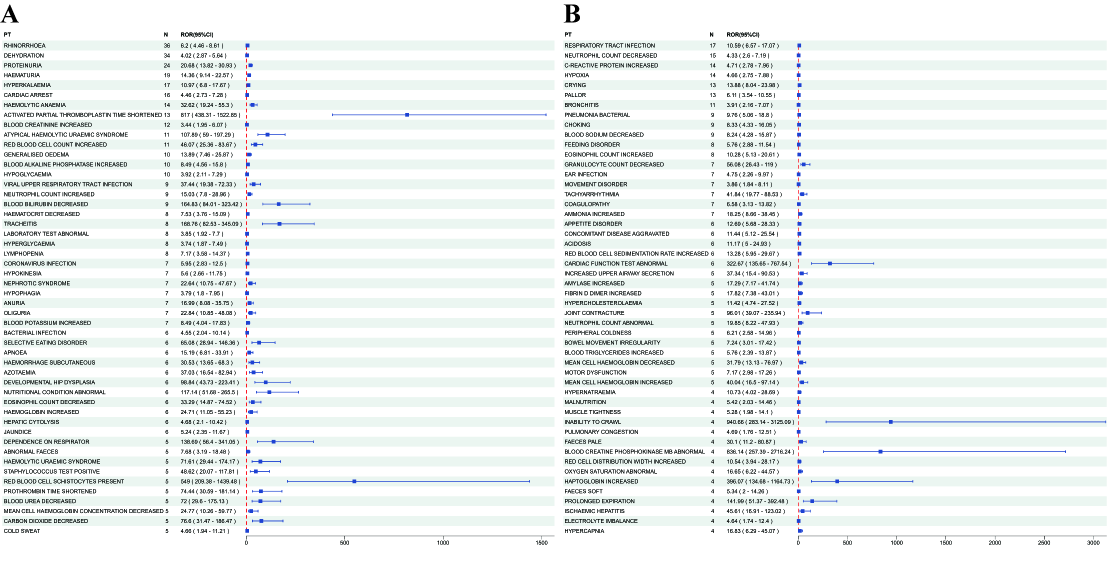


**Supplementary Figure S3. Sex Subgroup Analysis of Adverse Reactions Associated with Onasemnogene Abeparvovec (Zolgensma).**

1. Forest Plot of the Top 50 Female-Specific Adverse Events.
2. Forest Plot of the Top 50 Male-Specific Adverse Events.

Analyses were conducted using sex subgroups to identify adverse reactions that met the criteria of all four algorithms. Subsequent comparative analyses within each subgroup were performed to select adverse reactions specific to each subgroup and to screen for Preferred Terms (PTs) with a significance level of P<0.01 for presentation. If the number of subgroup-specific adverse reactions exceeded 50, the top 50 PTs were selected for reporting.

A total of 110 specific adverse events were identified for the female subgroup, and 87 for the male subgroup.

**Abbreviations**: PT, Preferred Term; N, Number of Reports of Adverse Drug Reactions; ROR(95%CI), Reporting Odds Ratio (ROR) with a 95% Confidence Interval.


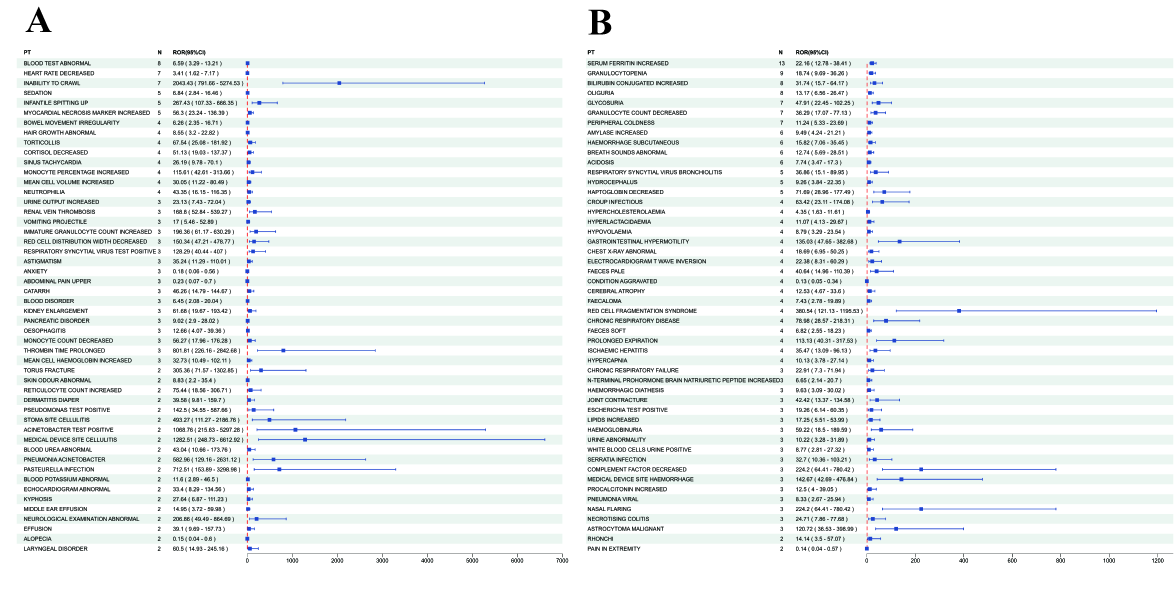


**Supplementary Figure S4. Subgroup Analysis of Adverse Reactions Associated with Onasemnogene Abeparvovec (Zolgensma) by Reporter Type.**

1. Forest Plot of Consumer-Reported Specific Adverse Events.
2. Forest Plot of Physician-Reported Specific Adverse Events.

Analyses were conducted using reported person subgroups to identify adverse reactions that met the criteria of all four algorithms. Comparative analyses within each subgroup were performed to select adverse reactions specific to each subgroup and to screen for Preferred Terms (PTs) with a significance level of P<0.01 for presentation. If the number of subgroup-specific adverse reactions exceeded 50, the top 50 PTs were selected for reporting.

A total of 137 specific adverse events were reported by consumers, and 156 specific adverse events were reported by physicians.

**Abbreviations**: PT, Preferred Term; N, Number of Reports of Adverse Drug Reactions; ROR(95%CI), Reporting Odds Ratio (ROR) with a 95% Confidence Interval.


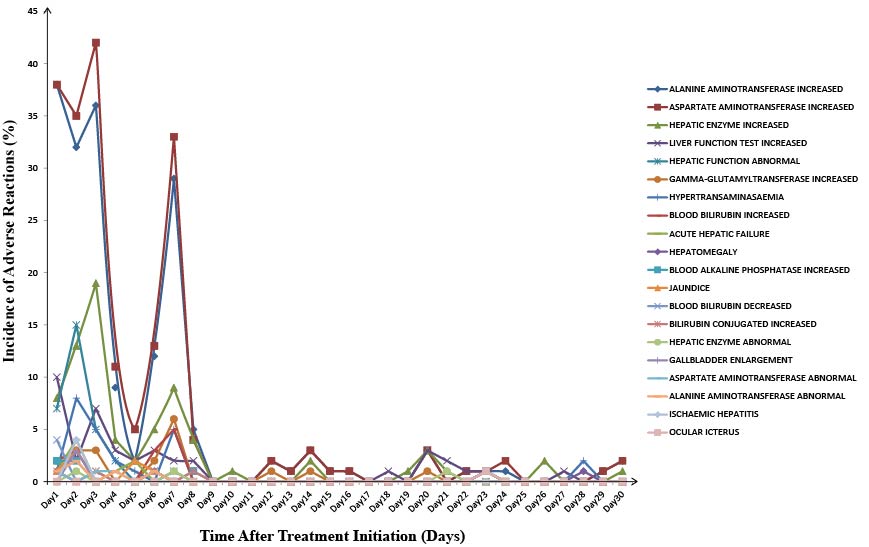


**Supplementary Figure S5. Temporal Distribution of Liver Function-Related Adverse Reactions Following Zolgensma Administration Within One Month.**

This statistical chart illustrates the occurrence of adverse reactions associated with liver function within the first month after Zolgensma treatment. The data reveal a notable concentration of hepatic function-related adverse events within the initial eight days post-administration, with subsequent smaller peaks observed on the 14th and 20th days. These findings suggest a potential early onset of liver-related adverse reactions, which may warrant further investigation into the temporal patterns of Zolgensma's safety profile.
